# Rapid implementation of an emergency on-site CKRT dialysate production system during the COVID-19 pandemic

**DOI:** 10.1186/s12882-023-03260-9

**Published:** 2023-08-22

**Authors:** J. Pedro Teixeira, Lisa Saa, Kevin A. Kaucher, Ruben D. Villanueva, Michelle Shieh, Crystal R. Baca, Brittany Harmon, Zanna J. Owen, Ismael Mendez Majalca, Darren W. Schmidt, Namita Singh, Saeed K. Shaffi, Zhi Q. Xu, Thomas Roha, Jessica A. Mitchell, Sevag Demirjian, Christos P. Argyropoulos

**Affiliations:** 1 Division of Nephrology, University of New Mexico (UNM) School of Medicine, MSC10-5550, 1 University of New Mexico, Albuquerque, NM 87131 USA; 2 Center for Adult Critical Care, UNM Hospital, Albuquerque, NM USA; 3 Acute Dialysis and CRRT Program, UNM Hospital, Albuquerque, NM USA; 4 Department of Internal Medicine, UNM School of Medicine, Albuquerque, NM USA; 5 Department of Pharmacy, UNM Hospital, Albuquerque, NM USA; 6 Department of Emergency Medicine, UNM School of Medicine, Albuquerque, NM USA; 7 Department of Nephrology and Hypertension, Cleveland Clinic, Cleveland, OH USA

**Keywords:** CRRT, CKRT, COVID-19 surge, Pandemic preparedness

## Abstract

**Background:**

On December 29, 2021, during the delta wave of the Coronavirus Disease 2019 (COVID-19) pandemic, the stock of premanufactured solutions used for continuous kidney replacement therapy (CKRT) at the University of New Mexico Hospital (UNMH) was nearly exhausted with no resupply anticipated due to supply chain disruptions. Within hours, a backup plan, devised and tested 18 months prior, to locally produce CKRT dialysate was implemented. This report describes the emergency implementation and outcomes of this on-site CKRT dialysate production system.

**Methods:**

This is a single-center retrospective case series and narrative report describing and reporting the outcomes of the implementation of an on-site CKRT dialysate production system. All adults treated with locally produced CKRT dialysate in December 2021 and January 2022 at UNMH were included. CKRT dialysate was produced locally using intermittent hemodialysis machines, hemodialysis concentrate, sterile parenteral nutrition bags, and connectors made of 3-D printed biocompatible rigid material. Outcomes analyzed included dialysate testing for composition and microbiologic contamination, CKRT prescription components, patient mortality, sequential organ failure assessment (SOFA) scores, and catheter-associated bloodstream infections (CLABSIs).

**Results:**

Over 13 days, 22 patients were treated with 3,645 L of locally produced dialysate with a mean dose of 20.0 mL/kg/h. Fluid sample testing at 48 h revealed appropriate electrolyte composition and endotoxin levels and bacterial colony counts at or below the lower limit of detection. No CLABSIs occurred within 7 days of exposure to locally produced dialysate. In-hospital mortality was 81.8% and 28-day mortality was 68.2%, though illness severity was high, with a mean SOFA score of 14.5.

**Conclusions:**

Though producing CKRT fluid with IHD machines is not novel, this report represents the first description of the rapid and successful implementation of a backup plan for local CKRT dialysate production at a large academic medical center in the U.S. during the COVID-19 pandemic. Though conclusions are limited by the retrospective design and limited sample size of our analysis, our experience could serve as a guide for other centers navigating similar severe supply constraints in the future.

**Supplementary Information:**

The online version contains supplementary material available at 10.1186/s12882-023-03260-9.

## Introduction

The Coronavirus Disease 2019 (COVID-19) pandemic has profoundly impacted healthcare delivery worldwide. The combination of the immense strain of extreme patient volumes and repeated pandemic-related disruptions in vital supply chains has posed major challenges to healthcare systems, including challenges in the ability to provide kidney replacement therapy (KRT) [1]. COVID-19 is associated with high rates of acute kidney injury (AKI) and need for KRT [2, 3]. At the peak of the pandemic, the demand for KRT machines increased 279% over baseline, placing a tremendous strain on hospital systems and their ability to provide such therapy [4–6]. Unlike pandemic spread, which can be modeled and predicted to an extent, supply chain disruptions are potentially more unpredictable and challenging to prepare for or anticipate.

At the University of New Mexico Hospital (UNMH), these challenges culminated in late December 2021, as the delta wave of the COVID-19 pandemic was transitioning to the omicron wave in New Mexico [7]. On the morning of December 29, 2021, the nephrology and critical care providers at UNMH were informed that our local supply of continuous kidney replacement therapy (CKRT) solutions was nearly exhausted and, due to pandemic-related disruptions in the supply chain, additional supplies were not anticipated. Within hours, the nephrology division implemented a backup plan (devised and tested approximately 18 months prior) to produce CKRT dialysate using the hospital water supply and locally available equipment and supplies. This program allowed us to sustain our CKRT program for approximately two weeks until the supply of premanufactured CKRT solutions was restored. In this report, we describe the development, implementation, and outcomes of this emergency on-site CKRT dialysate production system.

## Methods

### Design and setting

This is a single-center retrospective case series and narrative report describing and reporting the outcomes of the implementation of an on-site CKRT dialysate production system. UNMH is a publicly funded, tertiary care center serving as the primary safety net hospital for the state of New Mexico, providing care to a largely medically underserved population [8]. It has 618 licensed beds, including 72 beds in three adult intensive care units (ICUs), though the ICUs and the hospital as a whole have been consistently operating above their licensed capacity since early in the pandemic. As of 2022, approximately 250 patients are treated with CKRT over approximately 30,000 therapy hours each year at UNMH. Our CKRT normally utilizes PrismaSol and PrismaSATE solutions delivered by Prismaflex or PrisMax devices with M100 or HF1000 hemofilters (all from Baxter International, Deerfield, IL). Approval to conduct the study with waiver of informed consent was obtained from the UNM Health Sciences Human Research Protections Program (protocol #22–211). The Strengthening the Reporting of Observational studies in Epidemiology (STROBE) checklist for cohort studies [9] was followed whenever applicable.

### Patient population

All adults treated with locally produced CKRT dialysate were identified using fluid inventory records generated during the period of program operation, December 30, 2021, to January 11, 2022.

### Outcomes and data sources

Outcomes include results of testing of dialysate samples at 24 and 48 h after fluid production, rates of catheter-associated bloodstream infection (CLABSI) within 7 and 28 days of exposure to locally produced dialysate, and in-hospital mortality and mortality within 28 days of exposure.

Testing of CKRT dialysate samples for composition and microbiologic contamination was performed using the same procedures used for surveillance of our acute dialysis program, including shipping specimens at room temperature to a reference laboratory [Spectra Laboratories, Southaven, MS] where testing occurs approximately 24 h after collection. Endotoxin units (EUs) were measured using a kinetic quantitative chromogenic test and 48-h colony forming units (CFUs) were measured on trypticase soy agar using the heterotrophic plate count method, with lower limits of detection of 0.010 EU/mL and 2 CFUs/mL, respectively.

CLABSI cases were independently identified by our institutional healthcare-associated infection surveillance program and corroborated with the electronic health record (EHR). All other data were obtained from the EHR and fluid inventory records, with follow-up performed through the end of each index admission.

Given the high mortality associated with AKI requiring KRT in the ICU [10–13], especially in patients with COVID-19 [2, 3, 14, 15], we sought to capture disease severity by calculating Sequential Organ Failure Assessment (SOFA) scores [16]. The variables used to compute the SOFA scores included the most abnormal values from the 24-h period prior to exposure to locally produced CKRT dialysate whenever available.

### Statistical analysis

Continuous variables were tested for normality by histogram visualization and the Shapiro–Wilk test. Continuous variables with normal distribution are presented as mean ± standard deviation whereas those that are not normally distributed are presented as median [interquartile range]. Categorical variables are presented as frequencies and proportions. Data compilation and statistical analyses were performed using Excel (Microsoft, Redmond, WA) and R version 4.2.2 (R Foundation for Statistical Computing, Vienna, Austria).

## Results

### Plan development, fluid production trial, and fluid testing

**Fig. 1:**
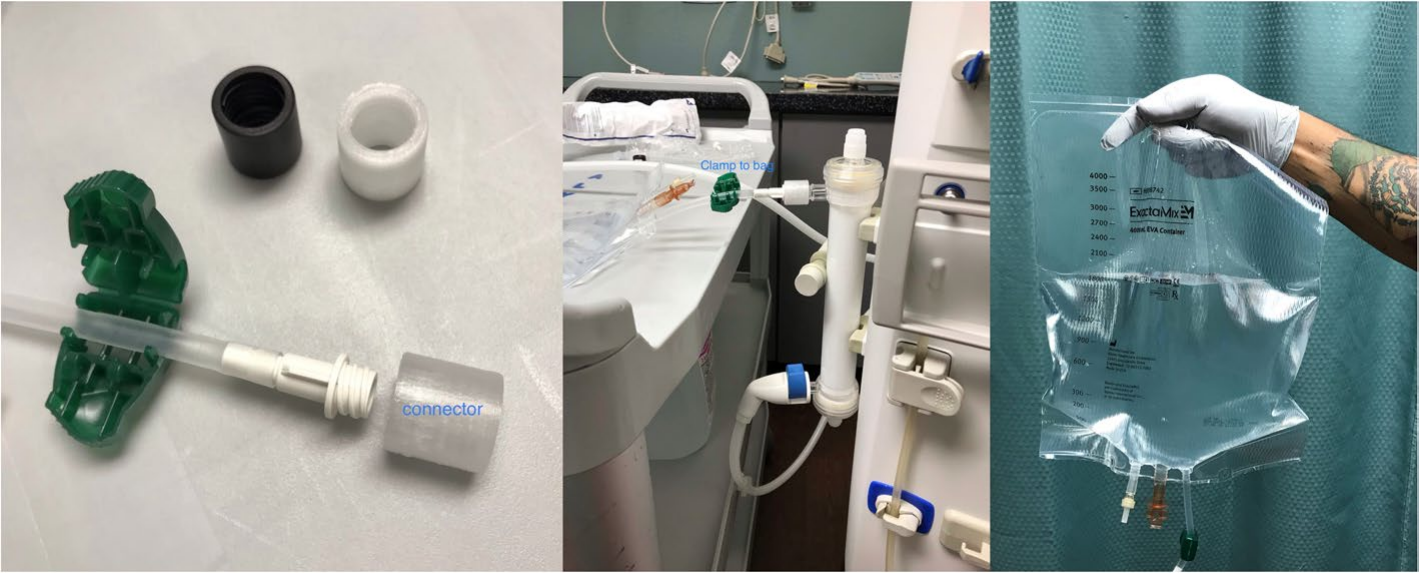
Trial phase of on-site CKRT dialysate production system. During the testing phase in July 2020, we procured prototypes of 3-D printed biocompatible connectors (**A**) from a local contractor. Using these connectors along with the IHD machines, dialyzers, and hemodialysis concentrate (**B**) that we typically use in our adult inpatient dialysis program and sterile bags normally utilized for parenteral nutrition (**C**), we generated bags of CKRT dialysate to both trial the system and to perform testing for fluid composition and microbiologic contamination. Abbreviations: CKRT, continuous kidney replacement therapy; IHD, intermittent hemodialysis

**Fig. 2:**
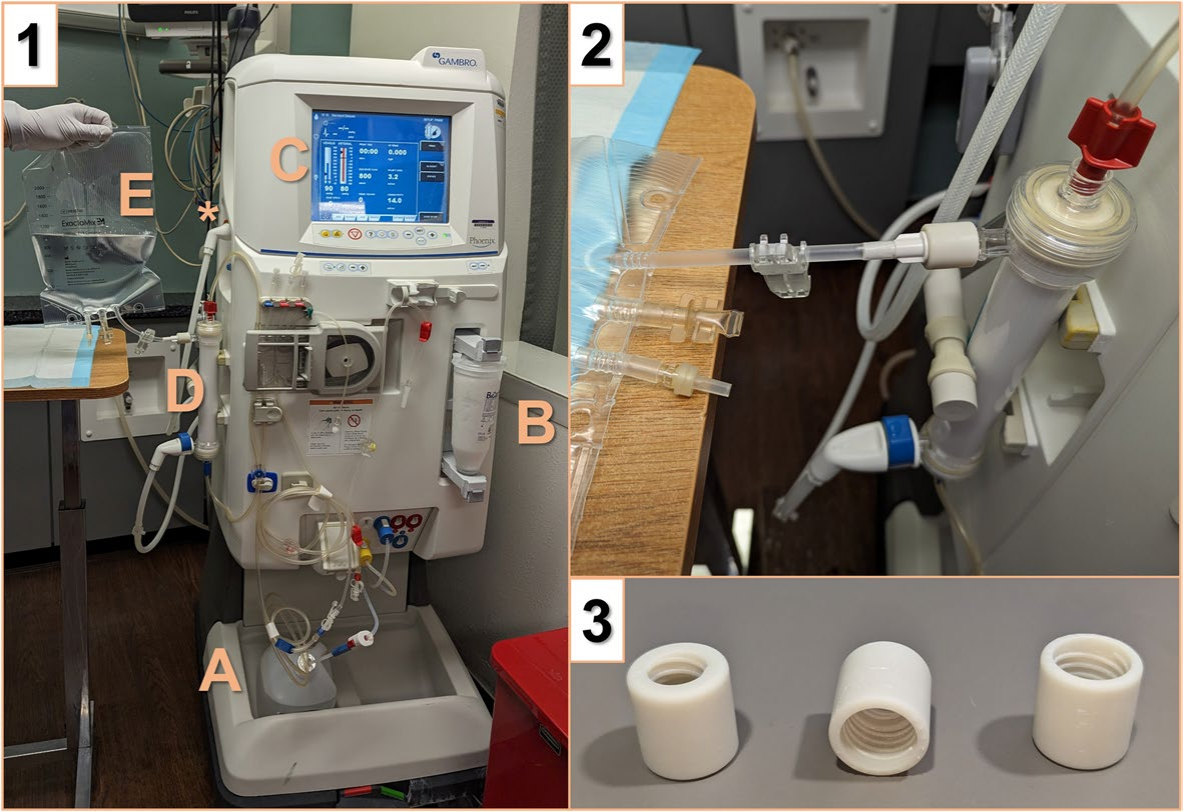
Full Circuit used for CKRT Dialysate Production. To illustrate the equipment used in better detail, we staged an additional session of CKRT dialysate production after the pandemic (Panel 1). Components of the circuit include (**A**) a liquid hemodialysis acid concentrate source (Naturalyte, Fresenius), (**B**) a powered bicarbonate concentrate source (BiCart, Baxter), (**C**) an IHD machine (Gambro Phoenix, Baxter) programmed to run in standard mode with dialysate flow rate of 800 mL/min and blood pump speed of 0, and (**D**) a standard IHD dialyzer (Revaclear, Baxter) connected through the inlet dialysate port to the clean dialysate supply and connected (Panel 2) by the effluent port via an adaptor to (**E**) a sterile TPN bag (Exactamix Empty EVA Bag, Baxter). To prevent the device from alarming or automatically entering bypass mode, an unprimed cartridge blood set is applied and the dialysate waste line (i.e., red dialysate connector, asterisk in Panel 1) is disengaged (though it is loosely attached to the machine in this image). The connectors (Panel 3) used to connect the TPN bags in the implementation phase of our CKRT dialysate production system are composed of biocompatible rigid material (“Figure 4 MED-WHT 10”, 3D Systems) and – though non-sterile – are designed specifically to not come into contact with the dialysate fluid. Abbreviations: CKRT, continuous kidney replacement therapy; IHD, intermittent hemodialysis; TPN, total parenteral nutrition

In July 2020, the nephrology division at UNM developed a backup plan for CKRT provision using locally generated fluid based largely on protocols previously published in the medical literature [17, 18] and online [19] early in the pandemic. The equipment and supplies used included traditional intermittent hemodialysis (IHD) machines (Gambro Phoenix, Baxter), Revaclear dialyzers (Baxter), Naturalyte hemodialysis acid concentrate (Fresenius Medical Care, Waltham, MA), BiCart powdered sodium bicarbonate concentrate (Baxter), and sterile 3-L and 4-L bags (Exactamix Empty EVA Bag, Baxter) normally used for total parenteral nutrition (TPN) (Figs. 1 and 2). Connectors – non-sterile but designed to not come into contact with the dialysate – were made for purpose by a manufacturing contractor at Sandia National Laboratories (Albuquerque, NM) out of 3-D printed biocompatible rigid material (“Figure 4 MED-WHT 10”, 3D Systems, Rock Hill, SC). Testing of the fluid samples generated at this phase revealed appropriate electrolyte composition and undetectable bacterial growth and endotoxin levels (Table 1).

**Table 1:** Testing for composition and microbiologic contamination of locally produced CKRT dialysate

	**Trial Phase (July 2020)**		**Implementation Phase (January 2022)**	
Fluid Incubation Period (hours)^a^	0	0	24	48
Approximate Testing Interval (hours)^a^	24	48	48	72
Sodium (mEq/L)	140	141	142	143
Potassium (mEq/L)^b^	2.3	2.3	3.0	3.0
Chloride (mEq/L)	110	110	110	110
Calcium (mEq/L)	2.5	2.5	2.6	2.6
Bicarbonate (mEq/L)	34	33		
Magnesium (mEq/L)	1.0	1.0		
Glucose (mg/dL)	110	109		
Colony Count (CFU/mL)	< 2		< 2	2
Endotoxin Level (EU/mL)	< 0.010		< 0.010	< 0.010

*Abbreviations*: *CFU* colony forming units, *EU* endotoxin units

Conversion factors for units: glucose in mg/dL to μmol/L, × 0.05551; calcium or magnesium in mEq/L to mmol/L, × 0.5

^a^During the trial phase, the fluid was immediately collected after production and two samples were shipped at room temperature to the reference lab, one tested upon arrival approximately 24 h after collection and a second stored locally for 24 h at the lab and then tested approximately 48 h after collection; in contrast, in the implementation phase, dialysate bags were stored locally at room temperature for 24 h and 48 h, and then samples were collected from the bags and shipped to the reference lab and tested upon arrival approximately 24 h later

^b^We used 2K hemodialysis concentrate in the testing but used 3K concentrate in the implementation phase

### Program implementation

After being informed on the morning of December 29, 2021, that UNMH had less than a 24-h supply of premanufactured CKRT solutions, the on-service medical ICU and nephrology consult attendings, acute dialysis program medical and nursing directors, nephrology division leadership, UNMH ICU nursing leadership, and UNMH pharmacy leadership had a series of impromptu phone and text discussions about options for managing the shortage. After considering alternatives, such as converting entirely from CKRT to sustained low-efficiency dialysis (SLED) and the use of lactated Ringer’s solution as CKRT solution [20], the decision was made to implement the backup plan to locally generate CKRT fluid using IHD machines.

Additional dialysis unit staff were called into service. Three IHD machines in the pediatric inpatient hemodialysis unit, which is closer to the adult ICUs and typically runs at lower capacity than the adult hemodialysis unit, were commandeered. The first batch of dialysate was prepared and, after obtaining permission from hospital leadership and the UNMH COVID-19 Emergency Operations Committee, the first patient was transitioned from premanufactured CKRT solutions to locally produced CKRT dialysate later that afternoon. After observing the first patient for approximately an hour, the remaining six patients on CKRT were transitioned over to the new solutions. Given that our default CKRT prescription is usually continuous venovenous hemodiafiltration (CVVHDF) using approximately equal amounts of dialysate and replacement fluid, all patients were converted to prescriptions of equal total effluent dose using the locally produced fluid as dialysate only. As we follow the CKRT device manufacturer’s recommendation to infuse a minimum of 200 mL/h of fluid into the post-filter deaeration chamber to prevent clot formation in the chamber, we continued to run our machines in CVVHDF mode but with the bulk of therapy fluid run as dialysate and replacement fluid limited to 200 mL/h of post-filter saline or isotonic sodium bicarbonate. We opted, rather than adjusting the potassium concentration, to use our available supply of 3K hemodialysis concentrate and generate all CKRT dialysate with 3 mEq/L of potassium, unlike our default premanufactured solutions which have 4 mEq/L.

**Fig. 3:**
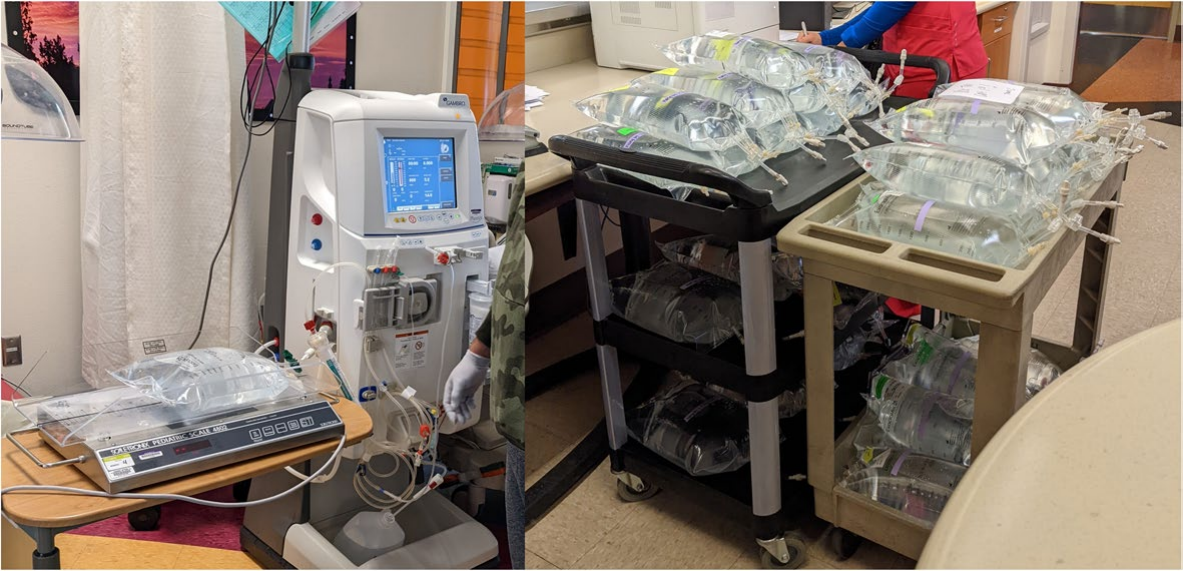
Implementation phase of on-site CKRT dialysate production system. During the implementation phase in December 2021 and January 2022, every morning our hemodialysis staff utilized two IHD machines in our pediatric hemodialysis unit to generate bags of CKRT dialysate (**A**). We developed a process for distributing the bags throughout our adult ICUs and a labeling system using colored tape and stickers to assist in tracking our inventory and to allow us to track the production date and time and the IHD machine of origin for every bag of dialysate produced (**B**). Photos were cropped for staff anonymity. Abbreviations: CKRT, continuous kidney replacement therapy; ICU, intensive care unit; IHD, intermittent hemodialysis

A logistical system was rapidly devised and implemented which included nightly fluid inventory, coordinated by the nephrology division and performed at approximately 8–10 pm by the nightshift charge nurses in the three adult ICUs, using usage sheets transmitted by secure email and spreadsheets maintained by the nephrology division and acute dialysis staff (see Additional files 1 and 2). Acute dialysis unit staff carried out CKRT dialysate production, labeling, and distribution starting every morning at approximately 4–6 am (Fig. 3). In addition to tracking shelf-life, the system permitted all bags to be traced back to the IHD machine that generated them, allowing for root-cause analysis in the case of any potential dialysate-related reactions.

### Fluid production and usage, patient characteristics, and CKRT prescription details

Over the next 13 days, 22 patients were treated with locally produced dialysate. See Tables 2 and 3 for summaries of the patient characteristics and CKRT prescriptions. Ultimately, 3,645 L of locally produced dialysate were utilized over a total of 2,024 CKRT treatment hours, corresponding to approximately 280 L of fluid utilized per day. The mean dose of dialysate utilized was 1800 mL/h, which, when including the 200 mL/h of post-filter saline or bicarbonate, resulted in a mean therapy fluid dose of 20.0 mL/kg/h or 28.0 mL/h per kg of ideal body weight. When analyzing all dose adjustments and considering each new dialysate dose as a new prescription, there were 55 distinct prescriptions with a median dose of 1800 [1300–2300] mL/h, corresponding to (when including the 200 mL/h of post-filter replacement fluid) a median prescribed dose of 21.6 [17.6–25.2] mL/kg/h or 28.8 [22.7–34.9] mL/h per kg of ideal body weight. Most patients underwent a single CKRT treatment run. Three patients were prescribed four runs of slow continuous ultrafiltration (SCUF) totaling 66 h, but all three were ultimately also treated with locally produced dialysate.

**Table 2:** Characteristics of the 22 patients treated with locally produced CKRT dialysate

	**No. (%) or Mean ± SD (Range)**
Age (years)	51.3 ± 12.5 (26–74)
Female gender	2 (9.1%)
Weight (kg)	100.1 ± 33.2 (50.2–182.5)
IBW (kg)	71.5 ± 7.9 (57–85)
Height (cm)	176.6 ± 8.5 (160–191)
BMI (kg/m^2^)	31.8 ± 9.5 (19.0–57.7)
Indication for CKRT	
AKI	15 (68.2%)
AKI on CKD	5 (22.7%)
Chronic kidney failure	2 (9.1%)
Primary Admission Diagnoses^a^	
COVID-19	11 (50%)
Sepsis/septic shock^b^	6 (27.3%)
Stroke or ICH	4 (18.2%)
Trauma/fall	3 (13.6%)
Liver disease^c^	2 (9.1%)
Overdose/intoxication	1 (4.5%)
Hemorrhagic shock	1 (4.5%)
Cardiac Arrest	1 (4.5%)
Rhabdomyolysis	1 (4.5%)
Seizure	1 (4.5%)
Illness Severity^d^	
SOFA score	14.5 ± 2.4 (9–19)
IMV	18 (81.8%)
NIV	1 (4.5%)
Vasopressor use	19 (86.4%)
Length of hospital stay (days)	31.4 ± 22.9

*Abbreviations*: *AKI* acute kidney injury, *BMI* body mass index, *CKD* chronic kidney disease, *CKRT* continuous kidney replacement therapy, *IBW* ideal body weight, *ICH* intracranial hemorrhage, *IMV* invasive mechanical ventilation, *NIV* non-invasive ventilation, *SD* standard deviation, *SOFA* sequential organ failure assessment

^a^Multiple diagnoses were present in some patients

^b^Excludes sepsis due to confirmed viral infection

^c^Includes end-stage liver disease and acute-on-chronic liver failure

^d^Data are from the 24 h prior to initial exposure to local dialysate whenever available; see Table 4 for additional details

**Table 3:** Prescription details of the CKRT delivered using locally produced dialysate

	**No. (%)**		**Mean ± SD or Median [IQR] (Range)**
Total no. of CKRT runs per patient^a^		**Mean Dose**^**c**^	
1	18 (81.8%)	**Dialysate (mL/h)**	1800
2	1 (4.5%)	**Total Therapy Fluid (mL/h)**^**d**^	2000
3	3 (13.6%)	**Total Therapy Fluid/ABW (mL/kg/h)**	20.0
Total no. of SCUF runs per patient^a^		**Total Therapy Fluid/IBW (mL/kg/h)**	28.0
0	19 (86.4%)		
1	2 (9.1%)	**Median Prescribed Dose**^**c**^	
2	1 (4.5%)	**Dialysate (mL/h)**	1800 [1300–2300] (1000–5000)
Anticoagulant Used^b^		**Total Therapy Fluid (mL/h)**^**d**^	2000 [1500–2500] (1200–5200)
None	12 (54.5%)	**Total Therapy Fluid/ABW (mL/kg/h)**	21.6 [17.6–25.2] (8.1–47.3)
Systemic unfractionated heparin (sUFH)	7 (31.8%)	**Total Therapy Fluid/IBW (mL/kg/h)**	28.8 [22.7–34.9] (14.1–69.3)
Prefilter (in-line) heparin	4 (18.2%)		
Regional citrate anticoagulation (RCA)	4 (18.2%)	**Duration of CKRT runs (hours)**^**a**^	55.7 [17.5–84.5] (4–312)
Simultaneous combined sUFH + RCA	4 (18.2%)		

*Additional Abbreviations*: *ABW* actual body weight, *CKRT* continuous kidney replacement therapy, *IBW* ideal body weight, *IQR* interquartile range, *SCUF* slow continuous ultrafiltration, *SD* standard deviation

^a^Treatment sessions in a patient separated by ≥ 6 h were considered separate CKRT or SCUF runs

^b^Includes all anticoagulant strategies used at any point; 6 patients were treated with 2 strategies and 1 treated with 4 different strategies; overall, at some point, 15 patients received heparin and 8 received citrate

^c^Mean dose was derived by taking the total volume of locally produced dialysate utilized and dividing by the total number of CKRT therapy patient-hours; median prescribed dose was derived by considering every dose adjustment as a new dose prescription, which yielded 55 distinct prescriptions

^d^Total therapy fluid includes locally produced dialysate and 200 mL/h of post-filter saline or isotonic sodium bicarbonate but excludes the contribution to total effluent dose of ultrafiltration performed for fluid removal

### Fluid testing for composition and microbiologic contamination

Initially, locally produced dialysate was used for a maximum of 24 h after production and then discarded. Random dialysate samples were obtained from bags that were stored locally for 24 and 48 h at room temperature and then shipped to the reference lab for testing 24 h later at 48 and 72 h (Table 1). In both cases, the testing revealed appropriate electrolyte composition, endotoxin level < 0.01 EU/mL, and colony count ≤ 2 CFU/mL. The endotoxin and culture results were well below the acceptable levels (< 2.0 EU/mL and < 200 CFU/mL, respectively) or actionable levels (≥ 1.0 EU/mL and ≥ 50 CFU/mL, respectively) per the standards set by the Association for the Advancement of Medical Instrumentation (AAMI) [21]. After reviewing these test results (and developing a relative shortage of TPN bags), we extended the shelf life of the solutions to 48 h to decrease waste. However, as we purposefully maintained a tight inventory with limited surplus, the vast majority of the fluid continued to be used within 24 h.

### Clinical outcomes

No CLABSIs occurred within 7 days of exposure to locally produced dialysate. When extending the search to within 28 days of exposure, a single case of CLABSI occurred in one patient 13 days after exposure. The mortality of the cohort was high, with an 81.8% in-hospital mortality and a 28-day mortality of 68.2%. As outlined in Tables 2 and 4, the illness severity of this cohort was also high. Of the 22 patients, 19 required vasopressor support and 18 required invasive mechanical ventilation in the 24 h prior to exposure to locally produced dialysate. The mean SOFA score, using data from the 24 h prior to exposure to locally produced dialysate whenever available, was 14.5 ± 2.3. Because of the change in default potassium concentration from 4 mEq/L in our premanufactured CKRT solutions to 3 mEq/L in our locally produced dialysate, we implemented a practice of checking serum chemistries a minimum of three times daily. However, as outlined in Table 5, the rates of hypokalemia were modest.

**Table 4:** SOFA score components for patients treated with locally produced CKRT dialysate

	**Mean ± SD or Median [IQR] (Range)**
SOFA score	14.5 ± 2.4 (9–19)
SOFA sub-scores	
Respiratory^a^	3.5 [2–4] (1–4)
Hematologic	0.0 [0–1] (0–3)
Neurologic	3.0 [2–4] (1–4)
Liver^b^	0.0 [0–2] (0–4)
Cardiovascular^c^	4.0[3, 4] (0–4)
Renal^d^	4.0 [3, 4] (2–4)
P/F (mmHg)^a^	100 [78.5–171] (42–319)
Platelet count (^a^10^9^/L)	185 [119–303] (40–643)
GCS	7 [3–10] (3–14)
Total bilirubin (mg/dL)^b^	1.0 [0.5–2.6] (0.2–28.5)
NED (mcg/kg/min)^c^	0.15 [0.05–0.33] (0–1.1)
Peak serum creatinine^d^	6.0 ± 2.3 (2.7–11.1)
Urine output (mL per 24 h)^d^	70.5 [0–280] (0–1051)

*Abbreviations*: *GCS* Glasgow coma scale, *IQR* interquartile range, *NED* norepinephrine equivalent dose, *P/F* ratio of partial pressure of oxygen in arterial blood to fraction of inspired oxygen, *SD* standard deviation, *SOFA* sequential organ failure assessment

Conversion factors for units: bilirubin in mg/dL to μmol/L, × 17.1; creatinine in mg/dL to μmol/L, × 88.4

^a^P/F ratio is reported for the 19 patients with arterial blood gas available within 48 h of starting CKRT; for 2 patients, the lowest SpO2/FiO2 ratio (rather than PaO2/FiO2 ratio) in the 24 h before exposure to local dialysate was utilized to determine the respiratory sub-score as per the modified SOFA score [22]; for 2 patients on nasal oxygen, FiO2 was estimated as 4% above room air per liter per minute of flow (e.g., 3 L/min = 21 + 4*3 = 33% FiO2)

^b^For patients with total bilirubin values not available in the 24-h period before exposure, the most recent value prior to exposure was utilized

^c^Norepinephrine equivalent doses of vasopressors were computed as proposed by Khanna et al. [23] to determine the cardiovascular sub-score

^d^Peak serum creatinine is reported for the 15 patients not yet on CKRT at the time of initial dialysate exposure; per local charting practices, urine output was tabulated over the 24-h period ending at 7 am prior to exposure

**Table 5:** Episodes of dyskalemia during treatment with locally produced CKRT dialysate

	**No. (%)**
Total no. of serum K levels obtained	460
Episodes of hyperkalemia (K ≥ 5.2 mEq/L)	65 (14.1%)
Episodes of hypokalemia (K ≤ 3.4 mEq/L)	39 (8.5%)
Mild hypokalemia (K 3.0–3.4 mEq/L)	33 (7.2%)
Severe hypokalemia (K ≤ 2.9 mEq/L)	6 (1.3%)
K = 2.9 mEq/L	5 (1.1%)
K = 2.7 mEq/L	1 (0.2%)

## Discussion

We describe the successful emergency implementation of an on-site CKRT dialysate production system in response to the unexpected disruption of our supply of premanufactured CKRT solutions during the COVID-19 pandemic. The keys to successful execution of this system included the foresight to develop a backup plan early in the pandemic, the flexibility of our acute dialysis program to shift manpower to the task of generating CKRT dialysate, and robust and frequent communication between the leadership of all institutional stakeholders in the CKRT program, including the nephrology division, acute dialysis program, critical care physicians and nursing, hospital pharmacy, and UNMH COVID-19 Emergency Operations Committee.

The use of premanufactured CKRT solutions, rather than locally compounded solutions, has been recommended when developing local KRT preparedness plans for pandemic-related surges to reduce the risk of compounding or nursing errors [24]. However, we ultimately had no viable alternative to sustain our CKRT program. Without this system, these 22 patients theoretically would have required transfer from UNMH to other regional hospitals. However, that likely would not have proven feasible given the limited bed capacity available in hospitals in the region at the time. For example, during this two-week period, UNMH ICU bed occupancy was at 114–132% of normal capacity while the general ward occupancy was at 125–148%. Moreover, hospital-to-hospital transfers throughout the region had largely ground to a halt during this period, leading many critically ill patients to board for days in the emergency departments of local access hospitals due to the lack of ICU beds at referral centers. As an illustration of the extreme disruption to normal referral patterns in the region, the medical intensivists at UNMH during this period received requests for transfer of ICU patients from as far away as Houston, TX, a much larger city over 750 miles away with vastly more medical resources than Albuquerque.

Using IHD machines to locally produce CKRT fluid is uncommon but not entirely novel [25–28]. The Cleveland Clinic, for example, has produced their own CKRT solutions using IHD machines for decades [26–28]. However, this report represents the first description of the rapid and successful implementation of a backup plan for on-site CKRT dialysate production program at a large academic medical center in the US during the COVID-19 pandemic. A large London center published a similar experience implementing in-house dialysate production during the COVID-19 pandemic, but the fluid in that setting was produced using pharmaceutically compounded solutions rather than using IHD machines [29]. Our facility did not have the pharmacy staffing capacity or supplies for such bulk compounding. Furthermore, using IHD machines to generate a single standardized CKRT solution from hemodialysis concentrate may introduce fewer opportunities for error than individually compounding dozens of bags of CKRT solution daily.

Though the mortality of this cohort was very high, the illness severity was proportionally high, with a median SOFA score of 14.5. SOFA scores above 14 have been shown to be associated with mortality rates of 80–95% [30, 31]. In addition, we previously documented similar mortality rates of AKI requiring CKRT at our institution earlier in the pandemic, when we were exclusively using premanufactured CKRT solutions. Specifically, we analyzed the outcomes of 67 patients with AKI treated with CKRT at UNMH during the pandemic in 2020 and found 30-day and in-hospital mortality rates of 63% and 72%, respectively, though disease severity was not tracked in that cohort [15]. Disease severity in this current cohort may have been especially high as we attempted to limit the use of the locally produced CKRT solution for multiple reasons, including the relatively limited track record of safety, a limited supply of TPN bags, and the production burden on our dialysis staff. As such, we managed as many patients as possible with IHD or SLED using vasopressor support, functionally reserving CKRT for the patients of highest illness severity.

Likewise, we attempted to limit fluid use by converting patients to SCUF when volume management alone was needed and by serially adjusting the dialysate doses downward as tolerated when solute control was adequate. Though CKRT doses below 20 mL/kg/h are not generally recommended, it has been suggested that such lower doses are reasonable in the setting of pandemic-related supply shortages [32–36]. Indeed, the two primary trials which have established the standard CKRT dose range of 20–25 mL/kg/h – which both demonstrated no benefit to higher doses of 35–40 mL/kg/h and excluded very obese patients – provide minimal insight into the safe lower dose limit for CKRT or the optimal approach to CKRT dose in obese patients [10, 11]. Observational studies from Japan [37, 38] suggest that CKRT doses of 15–20 mL/kg/h are likely safe, and such doses were used at other US centers during the pandemic [32, 33]. Similarly, in the absence of quality data, one proposed approach to CKRT dosing for patients of extreme weight is using adjusted or ideal body weight [36, 39]. We did – after starting patients on standard doses – successively lower doses in patients with adequate solute control on serial labs to decrease fluid usage. Moreover, given the mean BMI of our cohort was nearly 32 kg/m^2^, the median dose employed remained comfortably within the standard dose range if considering ideal body weight.

Our report has many limitations and caveats. First, the limited sample size of this cohort precludes any attempts to perform any meaningful statistical analyses of the outcomes. Furthermore, though the colony counts and endotoxin levels we report are reassuring, they do not prove that using in-house CKRT dialysate would have equivalent outcomes as sterile premanufactured solutions. For example, even low levels of endotoxin in dialysate fluid – levels still well within the AAMI standards – have been associated with increased mortality in observational data from the maintenance hemodialysis setting [40]. Notably, in the realm of CKRT, older data suggest that microbiologic or endotoxin contamination of CKRT fluid is more common than previously thought [41]. In addition, the recent ‘Regional citrate versus systemic heparin anticoagulation for continuous renal replacement therapy in critically ill patients with acute kidney injury (RICH) trial’, which randomized nearly 600 CKRT patients to the use of regional citrate versus systemic heparin, also suggested that the baseline risk of infection associated with CKRT may be higher than previously appreciated [42]. In the RICH trial, though citrate proved more effective at prolonging filter life, the citrate group had a surprisingly higher rate of new infections than the heparin group. On post-hoc analysis, the difference appeared attributable to increased filter lifespan, with an increase in filter lifespan of 10 h associated with a 21% higher risk of infection, though the authors speculated the mechanism was unlikely to be microbiologic contamination [42, 43]. Regardless, additional serial testing of this on-site fluid production system would be required to confirm acceptable purity before routinely using such fluid to support our CKRT program.

Our system also had important logistic limitations. We opted to utilize TPN bags for our locally produced CKRT solutions as TPN bags, though smaller than bags utilized for peritoneal dialysis, are designed to be filled in a sterile manner and then re-accessed in a sterile manner through a separate access port. However, we rapidly encountered a relative shortage of TPN bags, which we were suddenly using at a much higher rate than normal as an institution, ultimately requiring us to procure additional bags from other hospitals in the area. To reduce bag wastage, we ultimately extended the shelf life of our CKRT solutions up to 48 h after confirming reassuring endotoxin and bacterial culture results at 48 h. In addition, we did not have access to Y-connectors utilized by others [17, 28]. but rather connected one bag to each IHD machine at a time. As a result, our system was labor intensive and would not have been possible without multiple acute dialysis staff members working significant overtime on short notice. Sustaining such a program beyond two weeks would have required significant changes to our acute dialysis program staffing model.

Moreover, the CKRT dialysate generated using IHD machines must not be used as replacement fluid to perform hemofiltration, as the resulting solution (though “pure”) is technically not sterile, is not regulated by the FDA as a medication, and therefore may not be directly infused into patients [41]. However, the clinical relevance of this limitation is likely minimal, given the lack of evidence for any difference in clinical outcomes when using hemofiltration instead of hemodialysis [44]. In addition, though adjustments to the hemodialysis concentrate are theoretically possible, our streamlined system did not permit modification of the CKRT fluid composition. However, episodes of hypokalemia were less frequent than anticipated, possibly because of the purposefully lower CKRT doses utilized. Furthermore, though we normally use calcium-free solutions with citrate, we continued to employ regional citrate anticoagulation with this dialysate as use of calcium-containing solutions with citrate has previously been shown to be feasible and safe [45].

Finally, the use of IHD machines to generate CKRT dialysate is not an FDA approved procedure. Ultimately, we agree with the recommendation that centers that normally utilize premanufactured CKRT solutions should continue whenever possible to use such solutions during pandemic-related surges in patient volumes to minimize risk of errors [24].

However, despite these notable caveats, this system allowed us to sustain our CKRT program for nearly two weeks despite the unexpected disruption in our supply of premanufactured CKRT solutions during a period in the pandemic when transferring these 22 extremely ill patients would have likely not proven feasible. Though this report has many inherent limitations and the specifics of our program may not be fully generalizable to other centers, this detailed description of our experience could prove useful to other institutions navigating similar disruptions to their CKRT fluid supply in the future.

### Supplementary Information

**Additional file 1. **Inventory.xlsx: CKRT dialysate inventory example.

**Additional file 2. **Usage_sheet.docx: Daily MICU CRRT usage sheet.

## Data Availability

The datasets used in the current study are available from the corresponding author upon reasonable request.

## Abbreviations

AAMI: Association for the Advancement of Medical Instrumentation
AKI: Acute kidney injury
CLABSI: Catheter-associated bloodstream infection
CFUs: Colony forming units
CKRT: Continuous kidney replacement therapy
COVID-19: Coronavirus disease 2019
CVVHDF: Continuous venovenous hemodiafiltration
EHR: Electronic health record
EUs: Endotoxin units
ICU: Intensive care unit
IHD: Intermittent hemodialysis
KRT: Kidney replacement therapy
RICH trial: Regional citrate versus systemic heparin anticoagulation for continuous renal replacement therapy in critically ill patients with acute kidney injury trial
SOFA score: Sequential Organ Failure Assessment score
SCUF: Slow continuous ultrafiltration
SLED: Sustained low-efficiency dialysis
STROBE checklist: Strengthening the Reporting of Observational studies in Epidemiology checklist
TPN: Total parenteral nutrition
UNMH: University of New Mexico Hospital
