# Supplementary material for: Rapid implementation of an emergency on-site CKRT dialysate production system during the COVID-19 pandemic
Source: BMC Nephrol. 2023 Aug 22;24:245. doi: 10.1186/s12882-023-03260-9 (PMC10463836; doi:10.1186/s12882-023-03260-9)
Supplement: Supplementary file 2 — Additional file 2. Usage_sheet.docx: Daily MICU CRRT usage sheet. [file 12882_2023_3260_MOESM2_ESM.docx]

Additional_file_2_usage_sheet.docx: Daily MICU CRRT usage sheet

Description: An example from the medical ICU of the usage sheets filled out nightly by the charge nurse in each of the 3 adult ICUs and then transmitted by secure email to the CKRT dialysate workgroup, which consisted of acute dialysis unit staff and leadership and ICU nursing leadership.

**Complete between 2000 and 2200 daily. Please send via secure email to the CRRT workgroup using the email list below.**

[JTeixeira@salud.unm.edu](mailto:JTeixeira@salud.unm.edu); [XXXX@salud.unm.edu](mailto:XXXX@salud.unm.edu); [YYYY@salud.unm.edu](mailto:YYYY@salud.unm.edu); [ZZZZ@salud.unm.edu](mailto:ZZZZ@salud.unm.edu); [AAAA@salud.unm.edu](mailto:AAAA@salud.unm.edu); [BBBB@salud.unm.edu](mailto:BBBB@salud.unm.edu)

| RM# | MRN | Dialysate Rate |
| --- | --- | --- |
| MICU 6 | XXXXXXX | 1800 mL/hr |
| MICU 8 | XXXXXXX | 2000 mL/hr |
| MICU 12 | XXXXXXX | 5000 mL/hr |
| MICU 16 | XXXXXXX | 2000 mL/hr |
|  |  |  |
|  |  |  |
|  |  |  |
|  |  |  |
|  |  |  |
|  |  |  |
|  |  |  |
|  |  |  |
|  |  |  |
